# Iodine-123 Metaiodobenzylguanidine (I-123 MIBG) in Clinical Applications: A Comprehensive Review

**DOI:** 10.3390/ph17121563

**Published:** 2024-11-21

**Authors:** Ming-Cheng Chang, Cheng-Liang Peng, Chun-Tang Chen, Ying-Hsia Shih, Jyun-Hong Chen, Yi-Jou Tai, Ying-Cheng Chiang

**Affiliations:** 1 Department of Isotope Research Application, National Atomic Research Institute, Taoyuan 325207, Taiwan; mcchang@nari.org.tw (M.-C.C.); clpeng@nari.org.tw (C.-L.P.); ctchen@nari.org.tw (C.-T.C.); shihys@nari.org.tw (Y.-H.S.); mokon@nari.org.tw (J.-H.C.); 2 Department of Obstetrics and Gynecology, College of Medicine, National Taiwan University, Taipei 100233, Taiwan; 3 Department of Obstetrics and Gynecology, National Taiwan University Hospital, Taipei 100226, Taiwan; 4 Department of Obstetrics and Gynecology, National Taiwan University Hospital Hsin-Chu Branch, Hsin-Chu 302058, Taiwan

**Keywords:** I-123-MIBG, oncology, cardiology, neurology, clinical applications

## Abstract

Iodine-123 metaiodobenzylguanidine (I-123 MIBG) is a crucial radiopharmaceutical widely used in nuclear medicine for its diagnostic capabilities in both cardiology and oncology. This review aims to present a comprehensive evaluation of the clinical applications of I-123 MIBG, focusing on its use in diagnosing and managing various diseases. In cardiology, I-123 MIBG has proven invaluable in assessing cardiac sympathetic innervation, particularly in patients with heart failure, where it provides prognostic information that guides treatment strategies. In oncology, I-123 MIBG is primarily utilized for imaging neuroendocrine tumors, such as neuroblastoma and pheochromocytoma, where it offers high specificity and sensitivity in the detection of adrenergic tissue. Additionally, its role in neurology, specifically in differentiating between Parkinson’s disease, dementia, and Lewy body dementia, has become increasingly significant due to its ability to identify postganglionic sympathetic dysfunction. Despite its established clinical utility, the use of I-123 MIBG is not without limitations, including variability in imaging protocols and interpretation challenges. This review will explore these issues and discuss emerging alternatives, while also highlighting areas where I-123 MIBG continues to be a gold standard. By synthesizing the current research, this article aims to provide a clear understanding of the strengths, limitations, and prospects of I-123 MIBG in clinical practice.

## 1. Introduction

I-123 MIBG is a radiopharmaceutical that has had a profound impact on the field of nuclear medicine since its inception [1]. The development of MIBG dates back to the late 1970s, a period marked by significant advances in our understanding of the sympathetic nervous system and its associated pathologies [2]. MIBG was initially synthesized as an analog of guanethidine, a compound known for its ability to inhibit the release of norepinephrine [3]. The structural similarity of MIBG to norepinephrine allowed for it to be selectively taken up by adrenergic nerve terminals through the norepinephrine transporter, a characteristic that made it highly suitable for imaging the sympathetic nervous system [4,5]. The choice of Iodine-123 as the radionuclide for labeling MIBG was strategic. Iodine-123 has a half-life of approximately 13.2 h [6], which is long enough to perform diagnostic imaging but short enough to minimize the radiation exposure to the patient. Moreover, its gamma emission energy of 159 keV is ideal for imaging with gamma cameras, providing clear and detailed images that are essential for accurate diagnosis [6,7]. While the emission rate of the 159 keV gamma ray is 83.6%, the remaining intensity is carried by the conversion electrons. There are some other, but much weaker, gamma rays from the decay of I-123. The decay mode of I-123 is electron capture into the stable Te-123. Both the electron capture and conversion electron emission will create atomic vacancies, which will be followed by X-ray and Auger electron emission. According to the MIRD 2007 (Eckerman and Endo 2007) [8], on average 13.7 Auger electrons will be emitted in a single nuclear decay, and their average energy is less than 1 keV, which could have a significant impact in the vicinity of I-123 isotopes [9]. These properties, combined with the high specificity of MIBG for adrenergic tissues, positioned I-123 MIBG as a powerful tool in nuclear medicine, particularly for the imaging conditions associated with abnormal adrenergic function.

One of the earliest and most significant clinical applications of I-123 MIBG was in the diagnosis and management of neuroendocrine tumors, especially pheochromocytoma and neuroblastoma [10,11]. Pheochromocytoma, a tumor originating from the adrenal medulla, secretes catecholamines, leading to various clinical symptoms such as hypertension, palpitations, and headaches [12,13]. Neuroblastoma, on the other hand, is a pediatric malignancy arising from the sympathetic nervous system and is one of the most common extracranial solid tumors in children [14,15]. The ability of I-123 MIBG to localize adrenergic tumors with high specificity made it an invaluable tool in these contexts [16,17]. In neuroblastoma, I-123 MIBG scintigraphy became essential not only for initial tumor staging but also for evaluating the response to therapy and detecting recurrent disease [16]. Similarly, in pheochromocytoma, MIBG imaging plays crucial roles in the preoperative localization of the tumor, guiding surgical interventions, and providing prognostic information [18].

Beyond oncology, the application of I-123 MIBG has expanded significantly in cardiology, particularly in the assessment of cardiac sympathetic innervation [19]. The heart’s sympathetic nervous system plays a critical role in regulating cardiac function, and disturbances in this system are implicated in various cardiovascular diseases, most notably heart failure. In patients with heart failure, the sympathetic nervous system is often hyperactive, leading to a cascade of deleterious effects that worsen the patient’s condition. I-123 MIBG imaging allows for the non-invasive assessment of cardiac sympathetic nerve activity by quantifying MIBG uptake in the heart [20]. Reduced cardiac uptake of I-123 MIBG has been correlated with poor prognosis in heart failure patients, including higher risks of mortality and adverse cardiac events [21]. This prognostic capability makes I-123 MIBG an important tool in the management of heart failure, aiding in risk stratification and helping to tailor therapeutic strategies to individual patients [22]. The clinical importance of I-123 MIBG extends further into the realm of neurology, where it has become an important diagnostic tool in differentiating between PDD, LBD, and other forms of dementia, such as Alzheimer’s disease (AD) [23,24]. Both PDD and LBD are characterized by significant autonomic dysfunction, including the degeneration of sympathetic nerves in the heart. I-123 MIBG scintigraphy can visualize this cardiac sympathetic denervation, providing a distinctive imaging biomarker that helps differentiate these conditions from AD, which typically does not exhibit similar autonomic dysfunction [23,24]. This diagnostic capability is particularly valuable because PDD and LBD can present clinical symptoms that overlap with other dementias, making accurate diagnosis challenging. I-123 MIBG imaging thus offers a non-invasive method to improve diagnostic accuracy and guide appropriate clinical management [25]. Despite its widespread clinical utility, the use of I-123 MIBG is not without challenges. Variability in imaging protocols, differences in patient preparation, and variations in image interpretation across institutions can impact the consistency and reliability of results. Moreover, while I-123 MIBG remains a gold standard in certain diagnostic applications, the field of nuclear medicine is continuously evolving, with new radiopharmaceuticals and imaging modalities being developed. These advancements necessitate ongoing comparative studies to determine the relative merits of I-123 MIBG compared to the emerging alternatives and to refine its use in clinical practice [23]. In summary, I-123 MIBG represents a significant achievement in the field of nuclear medicine, offering unique capabilities in the diagnosis and management of a range of conditions across oncology, cardiology, and neurology. Its development and clinical integration have provided physicians with powerful tools to improve patient outcomes, although challenges in standardization and the advent of new technologies continue to shape its future role in medicine.

## 2. Radiopharmaceutical Characteristics of I-123 MIBG

**Figure 1:**
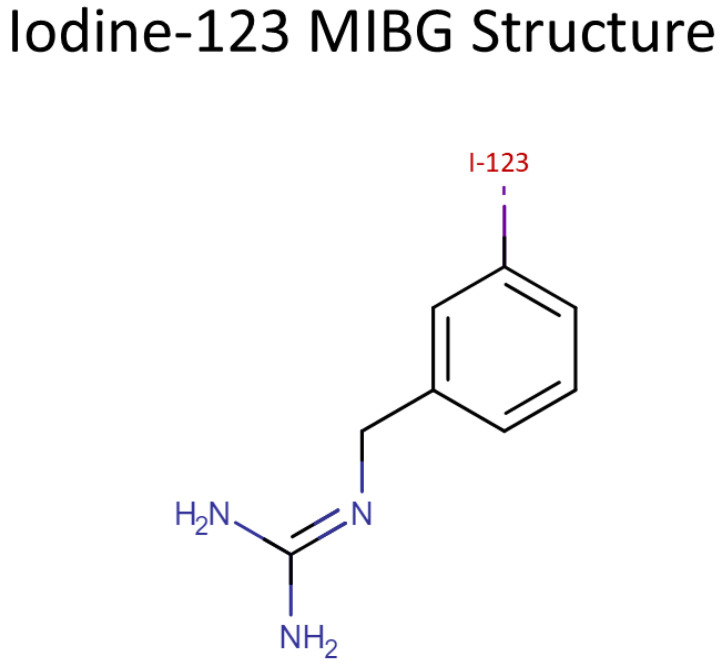
Iodine-123 MIBG structure. The structure of I-123 MIBG (iodine-123 metaiodobenzylguanidine) is shown, highlighting its radiolabeled composition. This compound is widely utilized in nuclear medicine for imaging neuroendocrine tumors and pheochromocytomas, owing to its affinity for adrenergic tissue. The isotope iodine-123 provides optimal imaging quality with minimal radiation exposure, making it a valuable tool in clinical diagnostics.

I-123 MIBG is a radiopharmaceutical that has become a cornerstone in nuclear medicine due to its unique properties that allow for the precise imaging of adrenergic tissues. The radiopharmaceutical is composed of a biologically active molecule, MIBG, which is chemically similar to the neurotransmitter norepinephrine [4], and the radioactive isotope Iodine-123 (Figure 1). This combination enables I-123 MIBG to be selectively taken up by adrenergic nerve terminals, making it particularly useful for imaging the sympathetic nervous system, as well as certain types of tumors and cardiac conditions. Understanding the radiochemical and pharmacokinetic properties of I-123 MIBG is essential for optimizing its clinical use.

### 2.1. Radiochemistry of I-123 MIBG

**Figure 2:**
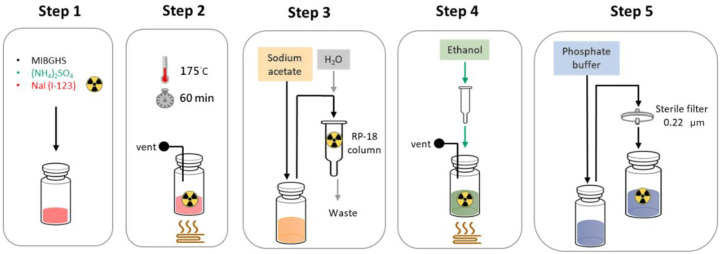
A semi-automated method for the radiosynthesis of I-123 MIBG. The five main steps of the I-123 MIBG radiolabeling procedure. This procedure includes 1. loading precursor and radioisotope; 2. Radiolabeling (heating the precursor to 175 °C for 60 mins for radioisotope labeling); 3. transfer and purification; 4. extraction and heating; and 5. dilution and sterile filtration.

The synthesis of I-123 MIBG involves the radioiodination of metaiodobenzylguanidine, a process that typically takes place in a radiopharmacy or specialized production facility [26]. The most common method for labeling MIBG with Iodine-123 is through electrophilic substitution, where the iodine atom is introduced into the aromatic ring of the MIBG. This is often accomplished by using oxidizing agents, such as chloramine-T or iodine monochloride, which facilitate the substitution of a hydrogen atom in the aromatic ring with the Iodine-123 isotope [27]. The labeling process begins with the preparation of the MIBG precursor, followed by the addition of Iodine-123 [27]. The five main steps of the I-123 MIBG radiolabeling procedure include (A) loading the precursor and radioisotope; (B) radiolabeling; (C) transfer and purification; (D) extraction and heating; and (E) dilution and sterile filtration on a semi-automated synthesizer (Figure 2). After the reaction is complete, the radiolabeled compound, I-123 MIBG, is purified to remove any unreacted iodine or by-products that may have formed during the synthesis. Purification is usually performed using high-performance liquid chromatography (HPLC) or other chromatographic techniques, which allow for the separation of I-123 MIBG from any impurities based on their chemical properties [28,29]. Once the radiolabeled MIBG has been purified, it undergoes rigorous quality control testing to ensure its safety and efficacy for clinical use. The key parameters that are assessed include radiochemical purity, specific activity, pH, sterility, and pyrogenicity. Radiochemical purity is particularly important, as it determines the proportion of the compound that is in the desired radiolabeled form versus any degraded or unreacted components. This is typically measured using thin-layer chromatography (TLC) or HPLC [29]. Specific activity refers to the radioactivity per unit mass of the compound and is crucial for ensuring that the administered dose delivers the appropriate amount of radiation to the target tissue. The pH of the solution is checked to ensure it is within a safe range for injection, while sterility and pyrogenicity tests confirm that the preparation is free from microbial contamination and fever-inducing substances, respectively.

### 2.2. Pharmacokinetics of I-123 MIBG

The pharmacokinetics of I-123 MIBG, including its distribution, metabolism, and excretion, play a critical role in its effectiveness as a diagnostic agent. After intravenous administration, I-123 MIBG is rapidly distributed throughout the body, with a particular affinity for tissues that are rich in adrenergic nerve endings, such as the heart, adrenal glands, and certain types of tumors [30].

#### 2.2.1. Distribution

The distribution of I-123 MIBG is primarily determined by its uptake into adrenergic neurons via the norepinephrine transporter (NET) [31]. This transporter is responsible for the reuptake of norepinephrine from the synaptic cleft into presynaptic nerve terminals, a process that I-123 MIBG mimics due to its structural similarity to norepinephrine. This uptake mechanism allows I-123 MIBG to concentrate in tissues with high adrenergic activity, making it a valuable tool for imaging sympathetic innervation in the heart and detecting neuroendocrine tumors. Once inside the adrenergic nerve terminals, I-123 MIBG is stored in synaptic vesicles, similar to endogenous norepinephrine. This sequestration within the vesicles helps to prolong the retention of I-123 MIBG in adrenergic tissues, which is beneficial for imaging purposes, as it allows for delayed imaging times that can provide clearer and more precise images.

#### 2.2.2. Metabolism and Excretion

I-123 MIBG is not significantly metabolized in the body, which contributes to its relatively straightforward pharmacokinetic profile. The compound remains largely intact as it circulates through the bloodstream and is taken up by adrenergic tissues [32]. This lack of metabolism is advantageous because it reduces the likelihood of forming radiolabeled metabolites that could interfere with imaging or result in unintended radiation exposure to non-target tissues. The primary route of excretion for I-123 MIBG is renal [33]. After administration, a portion of the injected dose is excreted unchanged in the urine, typically within the first 24 h. The rate of renal excretion can be influenced by factors such as renal function and the presence of any conditions that might affect renal clearance, such as dehydration or renal impairment [33,34]. It is important to monitor renal function in patients undergoing I-123 MIBG imaging, particularly in those with pre-existing renal conditions, as impaired renal clearance could lead to prolonged radiation exposure and potentially to an increased radiation dose to the kidneys and bladder [35].

#### 2.2.3. Factors Influencing I-123 MIBG Pharmacokinetics

Several factors can influence the pharmacokinetics of I-123 MIBG, affecting its distribution, uptake, and overall effectiveness as a diagnostic agent. The first factor to interrupt I-123 MIBG uptake is medications. Certain medications can interfere with the uptake of I-123 MIBG by adrenergic nerve terminals. Drugs that inhibit the norepinephrine transporter, such as tricyclic antidepressants, sympathomimetics, and certain antihypertensives, can reduce the uptake of I-123 MIBG, leading to less accurate imaging results [36,37,38]. It is generally recommended that such medications be discontinued prior to imaging to allow for an optimal uptake of I-123 MIBG. Next, the renal function of patients is also an important issue that influences I-123 MIBG imaging. As the primary route of excretion for I-123 MIBG is through the kidneys, impaired renal function can lead to reduced clearance of the compound from the body [35]. This can result in higher background radioactivity levels and prolonged exposure of non-target tissues to radiation, which may affect image quality and increase the radiation dose to the patient. Tumor burden is another issue for nuclear medicine physicians that might be considered. For patients with neuroendocrine tumors, the tumor burden can significantly influence the distribution of I-123 MIBG [17,36]. Large tumors with high adrenergic activity may sequester significant amounts of the radiopharmaceutical, potentially reducing its availability for uptake by other adrenergic tissues, such as the heart [36]. This can affect the accuracy of cardiac imaging in patients with extensive neuroendocrine tumors. In addition, age and gender are also important. Although less well-studied, factors such as age and gender may also influence the pharmacokinetics of I-123 MIBG [39]. For instance, age-related changes in adrenergic nerve function or renal clearance could potentially impact the distribution and excretion of the compound, while gender differences in body composition and hormonal regulation may also play a role [40].

## 3. Diagnostic Applications of I-123 MIBG

I-123 MIBG has established itself as a versatile and valuable radiopharmaceutical in clinical diagnostics, with significant applications across oncology, cardiology, and neurology [20,23,38]. Its ability to selectively target adrenergic tissues makes it particularly useful for imaging neuroendocrine tumors, assessing cardiac sympathetic innervation, and evaluating certain neurological disorders. I-123 MIBG has advantages over other iodine isotopes for medical imaging (Table 1). This section delves into the key clinical applications of I-123 MIBG, focusing on its role in neuroendocrine tumor imaging, cardiac imaging, and the diagnosis of neurological disorders.

### 3.1. Neuroendocrine Tumor Imaging

One of the most established uses of I-123 MIBG is in the imaging of neuroendocrine tumors, particularly pheochromocytomas, paragangliomas, and neuroblastomas [41,42]. These tumors originate from the cells of the sympathetic nervous system and have a high affinity for MIBG due to their adrenergic nature, making I-123 MIBG an ideal imaging agent.

#### 3.1.1. Applications in Pheochromocytomas and Paragangliomas

Pheochromocytomas are rare catecholamine-secreting tumors that arise from the adrenal medulla, while paragangliomas are similar tumors that originate outside the adrenal glands, typically in the paraganglia of the autonomic nervous system [43]. These tumors can cause significant clinical symptoms, including hypertension, headaches, palpitations, and sweating, due to the excessive production of catecholamines [44]. The accurate localization of these tumors is crucial for effective surgical planning and management. I-123 MIBG scintigraphy is highly effective in the detection and localization of pheochromocytomas and paragangliomas. The mechanism of action involves the uptake of I-123 MIBG by adrenergic nerve terminals within the tumor cells via the norepinephrine transporter. Once inside the cells, I-123 MIBG accumulates in the secretory vesicles, similar to endogenous catecholamines. This selective uptake allows for clear imaging of the tumor, distinguishing it from non-adrenergic tissues. The sensitivity and specificity of I-123 MIBG imaging for pheochromocytomas and paragangliomas are generally high, though they can vary depending on the size, location, and biological activity of the tumor [45,46]. In cases where the tumor expresses low levels of the norepinephrine transporter or has undergone dedifferentiation, the uptake of I-123 MIBG may be reduced, leading to false-negative results [47]. However, when positive, I-123 MIBG scans provide valuable information about tumor localization and extent, aiding in surgical decision-making and reducing the risk of incomplete resection. Additionally, I-123 MIBG scintigraphy is useful for identifying metastatic disease in patients with pheochromocytomas and paragangliomas [48,49]. These tumors can metastasize to various locations, including the liver, lungs, bones, and lymph nodes. The ability of I-123 MIBG to detect metastatic lesions makes it a critical tool for staging and guiding treatment strategies, which may include surgery, radiotherapy, or systemic therapies such as chemotherapy or targeted radionuclide therapy using Iodine-131-labeled MIBG [45].

#### 3.1.2. Applications in Neuroblastomas

**Figure 3:**
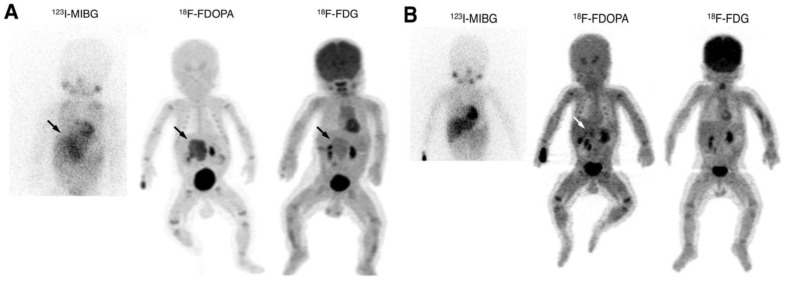
Comparison of I-123 MIBG, F-18 FDOPA PET, and F-18 FDG PET imaging modalities. (**A**) A 3-month-old boy diagnosed with stage-3 MYCN-nonamplified neuroblastoma initially exhibited positive uptake in I-123 MIBG, F-18 FDOPA PET, and F-18 FDG PET scans. Histopathological analysis of the right adrenal mass confirmed poorly differentiated neuroblastoma. (**B**) After completing three cycles of chemotherapy, the imaging findings showed a shift. The I-123 MIBG scan turned negative, while the F-18 FDOPA PET remained positive, and the F-18 FDG PET became negative. Subsequent tumor resection and histological analysis confirmed residual neuroblastic cells. The black and white arrows indicated the neuroblastoma location by I-123 MIBG, F-18 FDOPA or F-18 FDG imaging. This comparative analysis of PET imaging results builds upon the imaging framework previously outlined in our earlier publication [55].

Neuroblastoma is one of the most common pediatric malignancies, arising from the sympathetic nervous system, typically in the adrenal glands or along the sympathetic chain [50,51]. It accounts for a significant proportion of childhood cancers, particularly in infants and young children. Neuroblastomas are biologically diverse, with some tumors exhibiting aggressive behavior and a high likelihood of metastasis, while others may spontaneously regress. I-123 MIBG plays a central role in the diagnosis, staging, and management of neuroblastoma [52]. The ability of I-123 MIBG to target adrenergic tissue makes it highly effective in the visualization of neuroblastomas, which often express high levels of the norepinephrine transporter [53]. The radiopharmaceutical is used to perform whole-body scintigraphy, providing detailed information about the primary tumor site, as well as any metastatic spread to the bones, bone marrow, liver, or other organs (Figure 3) [54,55]. In the initial diagnosis of neuroblastoma, I-123 MIBG scintigraphy is used to assess the extent of the disease, which is critical for determining the stage and prognosis. The imaging results help guide treatment decisions, including the need for surgery, chemotherapy, radiation therapy, or a combination of these modalities. I-123 MIBG is also valuable in monitoring the response to therapy [41]. Following treatment, repeated MIBG scans can be performed to evaluate the effectiveness of the therapy and to detect any residual disease or recurrence [56]. One of the strengths of I-123 MIBG in neuroblastoma imaging is its ability to identify bone and bone marrow metastases, which are common in high-risk neuroblastoma and are associated with a poorer prognosis [57]. I-123 MIBG scintigraphy is often preferred over other imaging modalities, such as bone scintigraphy, because of its higher specificity for neuroblastoma and its ability to differentiate between neuroblastoma metastases and benign bone lesions. Despite its effectiveness, there are some limitations to I-123 MIBG imaging in neuroblastoma. For instance, not all neuroblastomas are MIBG-avid, and the absence of uptake on MIBG scans can occur in a subset of tumors, particularly for those that are poorly differentiated or have undergone neuroendocrine dedifferentiation. In such cases, alternative imaging modalities, such as PET/CT with F-18-FDG or Ga-68-DOTATATE, may be employed to complement the diagnostic workup [58,59,60].

### 3.2. Cardiac Imaging

I-123 MIBG has also found a significant role in cardiology, particularly in the assessment of cardiac sympathetic innervation. The autonomic nervous system, which includes the sympathetic and parasympathetic branches, plays a crucial role in regulating heart function [61]. Disruptions in sympathetic innervation can lead to various cardiovascular conditions, including heart failure and arrhythmias. I-123 MIBG imaging provides a non-invasive means of evaluating cardiac sympathetic activity, offering valuable insights into these conditions.

#### 3.2.1. Role of I-123 MIBG in Assessing Cardiac Sympathetic Innervation

Cardiac sympathetic innervation is essential for maintaining normal heart function, including regulating the heart rate, contractility, and vascular tone [62]. The sympathetic nerves release norepinephrine, which binds to adrenergic receptors on cardiac myocytes, leading to an increased heart rate and force of contraction [62]. In certain pathological conditions, such as heart failure, there is an imbalance in autonomic regulation, often characterized by heightened sympathetic activity and reduced parasympathetic activity [63]. This imbalance can contribute to disease progression and adverse outcomes. I-123 MIBG imaging is uniquely suited to assess cardiac sympathetic innervation because of its ability to mimic norepinephrine uptake and storage in sympathetic nerve terminals [64]. After intravenous administration, I-123 MIBG is taken up by the cardiac sympathetic nerves via the norepinephrine transporter and stored in synaptic vesicles [64]. The distribution and retention of I-123 MIBG in the heart reflect the integrity and function of the sympathetic nervous system. Cardiac I-123 MIBG scintigraphy involves acquiring planar or SPECT images of the heart at different time points after injection. The early images (usually taken 15–30 min post-injection) reflect the initial distribution of the radiopharmaceutical, while delayed images (taken 3–4 h post-injection) provide information about the retention and washout of I-123 MIBG [65]. The heart-to-mediastinum ratio (H/M ratio) and the washout rate are the key parameters used to quantify cardiac sympathetic innervation [66]. A lower H/M ratio and a higher washout rate indicate impaired sympathetic innervation and are associated with worse outcomes in heart failure patients [67,68].

#### 3.2.2. Applications in Heart Failure and Arrhythmias

Heart failure is a complex syndrome characterized by the inability of the heart to pump blood effectively, leading to symptoms such as shortness of breath, fatigue, and fluid retention. It is a leading cause of morbidity and mortality worldwide, and its management poses significant challenges. The autonomic nervous system plays a central role in the pathophysiology of heart failure, with increased sympathetic activity contributing to disease progression and adverse outcomes. I-123 MIBG imaging has emerged as a valuable tool in the risk stratification and management of heart failure [67,68]. Studies have shown that reduced cardiac uptake of I-123 MIBG, as indicated by a low H/M ratio, is associated with a higher risk of adverse outcomes, including mortality, sudden cardiac death, and hospitalization, due to heart failure exacerbations [69,70]. The washout rate of I-123 MIBG, which reflects the turnover of norepinephrine in sympathetic nerve terminals, is also a strong predictor of prognosis. A high washout rate indicates increased sympathetic nerve activity and is associated with poorer outcomes [64]. The prognostic value of I-123 MIBG imaging in heart failure has led to its incorporation into clinical practice guidelines in some regions, where it is used to help guide treatment decisions. For instance, I-123 MIBG imaging can identify patients who are at high risk for sudden cardiac death and may benefit from implantable cardioverter defibrillators (ICDs) [71,72,73]. It can also be used to monitor the response to pharmacological therapies, such as beta-blockers and angiotensin-converting enzyme (ACE) inhibitors, which are aimed at reducing sympathetic overactivity [74,75]. In addition to heart failure, I-123 MIBG imaging has applications in the evaluation of arrhythmias, particularly those associated with autonomic dysfunction. Cardiac arrhythmias, such as ventricular tachycardia and atrial fibrillation, can be driven by abnormal sympathetic activity [76]. I-123 MIBG imaging can help identify the regions of the heart with altered sympathetic innervation, which may be the focus of arrhythmogenic activity [77]. This information can be valuable in guiding therapeutic interventions, such as catheter ablation, to target the arrhythmogenic regions [77]. By identifying areas of the heart with impaired sympathetic innervation, clinicians can better understand the underlying mechanisms of arrhythmias and tailor treatment strategies to improve patient outcomes. Moreover, I-123 MIBG imaging is also being explored for its potential to predict the risk of arrhythmic events in patients with heart failure [68,71]. Patients with severe heart failure are at increased risk for sudden cardiac death due to ventricular arrhythmias. Identifying those at the highest risk is crucial for deciding on the implantation of an ICD, which can prevent sudden death by terminating life-threatening arrhythmias [72]. Studies have shown that patients with a low cardiac uptake of I-123 MIBG are more likely to experience arrhythmic events, making this imaging modality a valuable tool for risk assessment [63,64].

### 3.3. Neurological Disorders

In recent years, the utility of I-123 MIBG has extended beyond oncology and cardiology to include the evaluation of neurological disorders. The ability of I-123 MIBG to assess autonomic dysfunction, particularly in the sympathetic nervous system, has led to its use in diagnosing and differentiating neurodegenerative diseases, particularly Parkinson’s disease (PD) and related movement disorders, as well as emerging applications in dementia [78,79].

#### 3.3.1. Applications in Parkinson’s Disease and Related Movement Disorders

Parkinson’s Disease (PD) is a neurodegenerative disorder characterized by the progressive loss of dopaminergic neurons in the substantia nigra, leading to symptoms such as tremor, bradykinesia, rigidity, and postural instability. In addition to these motor symptoms, PD is often associated with autonomic dysfunction, including cardiovascular abnormalities like orthostatic hypotension and altered heart rate variability [80]. I-123 MIBG scintigraphy has proven to be a valuable tool in the evaluation of autonomic dysfunction in PD [81]. The radiopharmaceutical’s ability to assess cardiac sympathetic innervation allows clinicians to differentiate PD from other movement disorders that may present with similar clinical features. In PD, there is, typically, a marked reduction in the cardiac uptake of I-123 MIBG, reflecting the loss of sympathetic nerve endings in the heart [81]. This reduction is less pronounced or absent in other movement disorders, such as essential tremor or multiple system atrophy (MSA), making I-123 MIBG imaging a useful diagnostic tool [82,83]. The ability to differentiate PD from other neurodegenerative disorders is particularly important for guiding treatment strategies and providing patients with accurate prognostic information. For example, MSA, which can present with Parkinsonian symptoms similar to PD, often has a more rapid progression and poorer prognosis [83]. I-123 MIBG imaging can help distinguish these conditions, allowing for more tailored patient care. In addition to its diagnostic role, I-123 MIBG imaging is also being investigated for its potential to monitor disease progression in PD. By quantifying the degree of cardiac sympathetic denervation over time, clinicians may gain insights into the rate of disease progression and the effectiveness of therapeutic interventions [84,85]. While this application is still under investigation, it holds promise for enhancing the management of PD and improving patient outcomes.

#### 3.3.2. Applications in Dementia

Another emerging application of I-123 MIBG imaging is in the evaluation of dementia, particularly in distinguishing Lewy body dementia (LBD) from Alzheimer’s disease (AD) [79]. LBD is a common cause of dementia that shares many clinical features with AD, including cognitive decline, memory loss, and behavioral changes. However, LBD is also characterized by autonomic dysfunction and Parkinsonian features, which can complicate the diagnosis. I-123 MIBG scintigraphy offers a non-invasive method to differentiate LBD from AD by assessing cardiac sympathetic innervation [25,86]. Patients with LBD typically exhibit significant cardiac sympathetic denervation, as evidenced by a reduced cardiac uptake of I-123 MIBG [87]. In contrast, patients with AD usually have preserved cardiac sympathetic innervation, resulting in a normal or near-normal I-123 MIBG uptake. This distinction is crucial for accurate diagnosis, as the management and prognosis of LBD differ from those of AD. The use of I-123 MIBG for dementia diagnosis is still evolving, but it has the potential to become a standard component of the diagnostic workup for patients with suspected LBD [79,87,88]. By providing objective evidence of autonomic dysfunction, I-123 MIBG imaging can complement clinical assessments and other diagnostic tools, leading to more accurate diagnoses and better-informed treatment decisions.

## 4. Clinical Studies and Research on New Indications for I-123 MIBG

I-123 MIBG has been extensively studied across various clinical settings, particularly for neuroendocrine tumors, cardiac conditions, and emerging neurological applications. Key clinical trials have underscored the radiopharmaceutical’s diagnostic accuracy, prognostic value, and utility in guiding therapeutic interventions. For example, trials in neuroblastoma have established I-123 MIBG as the gold standard for staging and assessing treatment response, with studies demonstrating its superior specificity in the detection of metastatic disease compared to conventional imaging modalities [89,90]. Similarly, in cardiology, trials have highlighted the prognostic significance of I-123 MIBG uptake in heart failure, linking lower heart-to-mediastinum ratios to increased mortality and adverse cardiovascular events [91,92]. In neurology, clinical studies have explored the use of I-123 MIBG for differentiating PD from other movement disorders, with high sensitivity and specificity reported in the detection of cardiac sympathetic denervation associated with Parkinson’s dementia [81,93]. These studies have also demonstrated the potential of I-123 MIBG to identify LBD, aiding in its differentiation from AD. Summary of recent research on I-123 MIBG were shown in Table 2.

### 4.1. Comparative Analyses and Meta-Analyses

Comparative studies and meta-analyses have further validated the clinical utility of I-123 MIBG. For instance, meta-analyses of I-123 MIBG in neuroblastoma imaging have consistently shown its superior diagnostic performance compared to other imaging techniques, reinforcing its role in pediatric oncology [142]. In cardiology, comparative analyses have evaluated I-123 MIBG against other imaging modalities, such as PET and MRI, highlighting its unique ability to assess cardiac sympathetic function non-invasively [143]. These analyses have supported the integration of I-123 MIBG into clinical practice guidelines, particularly for risk stratification in heart failure. In neurology, meta-analyses have confirmed the high diagnostic accuracy of I-123 MIBG scintigraphy for distinguishing PD from other neurodegenerative disorders [82,144]. These studies suggest that I-123 MIBG could be a valuable addition to the diagnostic toolkit for movement disorders, potentially guiding more personalized treatment approaches.

### 4.2. Ongoing Research and Potential Areas for Exploration

The future of I-123 MIBG research is poised to expand beyond its established applications. Ongoing clinical trials are exploring its use in new therapeutic areas, such as in the assessment of autonomic dysfunction in diabetes and chronic kidney disease [145]. Additionally, research is investigating the role of I-123 MIBG in monitoring disease progression and therapeutic response in PD, which could lead to earlier intervention strategies and improved patient outcomes [146,147]. Another promising area of exploration is the application of I-123 MIBG in the early detection of neurodegenerative disorders beyond PD, such as multiple system atrophy and pure autonomic failure [82,84]. These conditions share similar autonomic dysfunctions, and I-123 MIBG imaging could offer a non-invasive means of diagnosing these disorders at an earlier stage. The development of new radiopharmaceuticals that build upon the success of I-123 MIBG is also a key focus of ongoing research. Innovations are aimed at enhancing the sensitivity and specificity of imaging agents, potentially leading to the development of next-generation radiopharmaceuticals with improved pharmacokinetics and reduced radiation exposure [81]. Additionally, research is exploring the combination of I-123 MIBG with other imaging modalities, such as PET/MRI hybrid imaging, to provide more comprehensive diagnostic information and improve clinical outcomes [60,86]. These future directions highlight the ongoing evolution of I-123 MIBG in clinical practice, with the potential to expand its utility into new areas of medicine and improve the management of a wide range of conditions. As research continues to advance, I-123 MIBG is likely to remain at the forefront of radiopharmaceutical innovation, offering new possibilities for diagnosis, prognosis, and therapy in both existing and emerging clinical applications.

## 5. Challenges to and Considerations of I-123 MIBG Imaging and Therapy

While I-123 MIBG is a highly valuable tool in nuclear medicine, it is not without limitations. One of the primary challenges in I-123 MIBG imaging is the variability in radiopharmaceutical uptake, which can be influenced by factors such as the patient’s medication use, tumor biology, and underlying health conditions. For example, certain medications, like tricyclic antidepressants, calcium channel blockers, and sympathomimetics, can interfere with the norepinephrine transporter, reducing the uptake of I-123 MIBG and leading to false-negative results [37,148,149]. Additionally, tumors with a low expression of the norepinephrine transporter may exhibit reduced I-123 MIBG uptake, complicating accurate imaging and diagnosis. Another challenge lies in the interpretation of I-123 MIBG images. Variability in imaging protocols, timing of scans, and differences in equipment and software can lead to inconsistencies in image quality and diagnostic accuracy across different institutions. Moreover, the presence of background noise and non-specific uptake can obscure the identification of lesions, particularly in the setting of small or early-stage tumors.

### 5.1. Strategies for Overcoming Limitations

To overcome these limitations, standardized imaging protocols and careful patient preparation are essential. Patients should be instructed to discontinue medications that may interfere with I-123 MIBG uptake prior to imaging. Consistent timing of imaging after radiopharmaceutical administration, typically at both early (15–30 min) and delayed (3–4 h) intervals, can help ensure reliable results [65]. Additionally, the use of advanced imaging techniques, such as SPECT/CT, can improve lesion detection and provide more accurate anatomical localization by combining functional and structural imaging. Enhancing the radiopharmaceutical’s affinity for adrenergic tissues through chemical modifications or the development of new tracers is another area of ongoing research that could mitigate some of the current challenges associated with I-123 MIBG. Further, incorporating artificial intelligence and machine learning into image analysis may help standardize interpretation and reduce variability across different centers [68,120].

### 5.2. Radiation Safety

Radiation safety is a critical consideration in the use of I-123 MIBG, particularly given its application in both diagnostic imaging and therapy. The dosimetry for I-123 MIBG must be carefully managed to minimize radiation exposure to patients while ensuring sufficient dose delivery to achieve accurate imaging or therapeutic outcomes. For diagnostic imaging, the radiation dose from I-123 MIBG is generally low and comparable to other nuclear medicine procedures. However, for therapeutic applications, particularly when using I-131 MIBG, the radiation dose can be significantly higher, requiring careful calculation to balance efficacy with safety. For healthcare providers, particularly those involved in the preparation and administration of I-123 MIBG, adherence to radiation safety protocols is paramount. This includes using shielding, maintaining an appropriate distance from radiation sources, and minimizing the exposure time. Personal dosimeters should be used to monitor cumulative radiation exposure, ensuring it remains within acceptable limits.

### 5.3. Guidelines and Best Practices

To safeguard both patients and healthcare providers, adherence to established guidelines and best practices is essential. The International Commission on Radiological Protection (ICRP) provides recommendations for radiation dose limits, which should be followed for all nuclear medicine procedures. For patients, individualized dosimetry calculations based on factors such as body weight, organ function, and tumor burden can help optimize the balance between diagnostic accuracy and radiation exposure. In clinical settings, the implementation of standard operating procedures (SOPs) for the handling and administration of I-123 MIBG is crucial. This includes proper training for healthcare personnel on radiation safety practices, the use of appropriate protective equipment, and regular audits of radiation safety protocols to ensure compliance. In summary, while I-123 MIBG is a valuable tool in nuclear medicine, addressing its limitations and ensuring rigorous radiation safety practices are key to maximizing its clinical utility and minimizing risks. Continued research and innovation in this field will further enhance the safety and efficacy of I-123 MIBG for both diagnostic and therapeutic applications.

## 6. Conclusions

I-123 MIBG has proven to be an invaluable radiopharmaceutical in the realm of nuclear medicine, offering significant diagnostic and therapeutic benefits across a variety of medical specialties. Its ability to selectively target adrenergic tissues has made it particularly effective for the imaging of neuroendocrine tumors, such as pheochromocytomas, paragangliomas, and neuroblastomas, as well as for assessing cardiac sympathetic innervation. In neurology, I-123 MIBG has emerged as a crucial tool for differentiating PD from other movement disorders and for distinguishing LBD from AD. Moreover, the therapeutic potential of I-131 MIBG, especially for the treatment of neuroendocrine tumors, highlights the synergistic use of I-123 and I-131 MIBG for both diagnosis and therapy. The clinical implications of I-123 MIBG are profound, as it continues to play a pivotal role in enhancing diagnostic accuracy, guiding therapeutic interventions, and improving patient outcomes. Its integration into clinical practice, particularly in oncology, cardiology, and neurology, underscores the importance of this radiopharmaceutical to modern medicine. However, addressing the limitations associated with I-123 MIBG, such as variability in uptake and image interpretation, remains essential. Strategies such as standardized imaging protocols, advanced imaging technologies, and innovations in radiopharmaceutical development are critical for overcoming these challenges. Looking forward, ongoing research into new applications for I-123 MIBG, as well as the development of next-generation radiopharmaceuticals, promises to expand its utility and effectiveness. The continued exploration of I-123 MIBG’s role in emerging clinical areas, combined with advancements in imaging techniques and personalized dosimetry, will likely enhance its impact on patient care. As these developments unfold, I-123 MIBG is expected to remain a cornerstone of nuclear medicine, contributing to more precise diagnostics, targeted therapies, and, ultimately, better clinical outcomes for a wide range of conditions.

## Figures and Tables

**Table 1:** Comparison of Iodine-123, Iodine-131, and Iodine-124 MIBG.

Feature	Iodine-123 MIBG	Iodine-131 MIBG	Iodine-124 MIBG
Purpose	Diagnostic imaging for nerve and heart health	Primarily treatment; some diagnostic use	PET imaging for cancer and molecular studies
Image Quality	High-resolution, clear images	Lower image quality, less clear	High-resolution PET images
Radiation Dose	Lower exposure, safer for repeated scans	Higher exposure, less ideal for frequent use	Higher radiation dose due to longer half-life
Half-Life	Shorter (13 h), with reduced radiation over time	Longer (8 days); stays in body longer	Longer (4.2 days); useful for tracking metabolic processes
Thyroid Safety	Lower risk, minimal need for blocking	Higher risk, requires thyroid-blocking medicine	Similar risk, may need thyroid-blocking medicine
Cost & Availability	More expensive, limited to specialized centers	Less expensive, more widely available	High cost, limited availability in PET centers
Primary Use Cases	Neurodegenerative and cardiac disease diagnosis	Treatment of certain cancers, limited cardiac diagnosis	PET imaging in cancer, long-term tracking
Patient Isolation	Not required after scan	May require temporary isolation after treatment	Not usually required, but depends on dose

**Table 2:** Summary of recent research on I-123 MIBG.

Study	Objective	Findings	Conclusions
Hirahara et al. 2024 [94]	Predict metastatic potential in pheochromocytoma (PHEO) and paraganglioma (PGL).	I-123 MIBG uptake scores and SUV-related parameters significantly differed between tumors with various adrenal gland scaled scores.	I-123 MIBG uptake assessment using SUV-related parameters can be an imaging tool for predicting metastatic potential in PHEO/PGL.
Shimasaki et al. 2024 [95]	Study the associations between homovanillic acid (HVA), striatal dopamine transporter (DAT), 5-hydroxyindole acetic acid (5-HIAA), and cardiac I-123 MIBG findings.	MIBG positivity was associated with 5-HIAA level. DAT-specific binding ratio correlated with both HVA and 5-HIAA. HVA was the only significant variable associated with Z-score of striatal DAT-specific binding ratio.	There are direct associations between 5-HIAA and cardiac MIBG and between HVA and striatal DAT binding.
Umehara et al. 2024 [96]	Investigate the associations between serum phosphorus levels and phenotypes and degeneration of cardiac sympathetic and nigrostriatal dopaminergic neurons in Parkinson’s disease (PD).	Serum phosphorus levels were significantly lower in PD with decreased heart-to-mediastinum (H/M) ratio than PD with normal H/M ratio. Lower serum phosphorus levels were significantly associated with more severe degeneration of nigrostriatal dopaminergic neurons.	Serum phosphorus levels and their association with nigrostriatal dopaminergic degeneration are different in patients with different H/M ratios. Serum phosphorus levels may reflect the degree of nigrostriatal dopaminergic degeneration in patients with decreased H/M ratio.
Nakajima et al. 2024 [97]	Determine the relationship between I-123 MIBG activity and lethal arrhythmic events (ArE) in Japanese and European cohorts.	Bell-shaped relationship in NYHA classes II/III, high washout rates, and ischemic etiology likely aid in identifying patients at high risk for lethal arrhythmic events.	The relationship between cardiac I-123 MIBG activity and ArE is influenced by the background of patients.
Hagen et al. 2024 [98]	Correlate echocardiographic parameters with quantitative reconstruction of I-123 MIBG uptake.	Absolute quantification of I-123 MIBG uptake in the LV and RV correlates accurately with the gold standard heart-to-mediastinum ratio.	Cardiac I-123 MIBG imaging using SUV facilitates image-guided therapy in patients diagnosed with arrhythmogenic right ventricular cardiomyopathy.
Borgwardt et al. 2024 [99]	Replace I-123 MIBG with new PET tracer 18F-MFBG.	Increased number of radiotracer-avid lesions, SIOPEN and Curie score 18F-MFBG LAFOV PET/CT compared with I-123 MIBG SPECT/CT.	18F-MFBG LAFOV PET/CT shows promise for future staging and response assessment of neuroblastoma.
Mizukami et al. 2024 [100]	Investigated I-123 MIBG and transcranial sonography (TCS) for diagnosis of high-signal-intensity substantia nigra lesion (HSI-SNL) incidence in Parkinson’s disease.	MIBG findings were normal in 27.3% with HSI-SNL and abnormal in 63.6% without HSI-SNL.	Multiple patients with normal findings during MIBG may have HSI-SNL. Confirmatory imaging of HSI-SNL with TCS may be useful for diagnosis.
Nakajima et al. 2024 [101]	Establish a diagnostic index for Lewy body diseases (LBD) and criteria for exempting patients from late imaging.	Early or late sympathetic MIBG (SMILe) index using a single heart-to-mediastinum ratio variable discriminated LBD from non-LBD. The AUC values for early and late SMILe indexes were 0.880 and 0.894. The sensitivity and specificity of early and late SMILe indexes were 76% and 90% and 76% and 87%, respectively.	SMILe index for LBD provides likelihood of LBD, and those with a 50% threshold demonstrated optimal diagnostic accuracy. The index values of either <0.3 or >0.7 accurately selected patients who do not need late imaging.
Mori et al. 2024 [102]	Compare diagnostic accuracy of whole-body MRI and I-123 MIBG in metastatic pheochromocytoma and paraganglioma.	Sensitivity, specificity, accuracy, PPV, NPV, and AUC were 82%, 97%, 90%, 96%, 86%, and 0.92 for WB-MRI images.	Comparable diagnostic accuracy of WB-MRI to I-123 MIBG images.
Saito et al. 2024 [103]	I-123 MIBG sympathetic nerve imaging with 3D quantitation to the diagnosis of neurological disorders.	Absolute heart counts and SUV determined using I-123 MIBG correlated with findings of conventional planar images in neurological diseases.	I-123 MIBG SPECT/CT imaging might differentiate patients with multiple system atrophy and progressive supranuclear palsy.
Miyamoto et al. 2024 [104]	Investigate whether cardiac postganglionic denervation is present in sleep behavior disorder and is a marker for Lewy body disease status.	Reduced I-123MIBG uptake was observed in the early and delayed images in 84.4 and 93.4% of patients with isolated rapid eye movement sleep behavior disorder.	Reduced I-123MIBG uptake is a robust maker for Lewy body disease among isolated rapid eye movement sleep behavior disorder patients.
Choi et al. 2024 [105]	Quantify I-123MIBG uptake in the adrenal glands using SPECT/CT images.	Labetalol inhibited the uptake of I-123 MIBG in cell lines expressing norepinephrine transporter.	SPECT/CT imaging with I-123 MIBG could be applicable for evaluating the preclinical efficacy of labetalol as a β-adrenergic receptor blocker.
Xavier de Brito et al. 2024 [106]	Investigate autonomic disorders with I-123 MIBG SPECT imaging in patients with long COVID.	75% of patients with symptoms of dysautonomia related to long COVID had reduced MIBG uptake, suggesting the presence of myocardial sympathetic denervation.	MIBG SPECT can be useful for the evaluation of the extent of myocardial sympathetic denervation; it provides quantitative data that aid the diagnosis of dysautonomia, determination of its prognosis, and evaluation of treatment effects.
Saito et al. 2024 [107]	Develop new methods for 3D heart segmentation and disease quantitation.	SPECT/CT WRs determined based on SUV closely correlated with planar heart-to-mediastinum ratios.	Sympathetic nervous activity could be absolutely quantified in 3D from I-123 SPECT/CT images.
Feng et al. 2024 [108]	Develop and validate an I-123 MIBG SPECT/CT-based radiomics nomogram and evaluate its value in predicting event-free survival.	The radiomics model predicted the best prognosis for event-free survival in both the training and validation cohorts, with C-indices of 0.819 and 0.712, and 1-year areas under the curve of 0.899 and 0.748, respectively.	I-123 MIBG SPECT/CT-based radiomics can accurately predict the event-free survival of high-risk neuroblastoma after induction of remission.
Ozawa et al. 2024 [109]	Study the relationship between sympathetic skin response (SSR), I-123 MIBG cardiac scintigraphy, and DaT scan imaging parameters in Parkinson’s disease (PD) and other Parkinsonian syndromes.	Multiple regression analyses showed a significant relationship between the amplitudes of SSR and DaT scan imaging in PD.	Unlike multiple system atrophy and progressive supranuclear palsy, sudomotor nervous system is parallelly involved with cardiac sympathetic and central dopaminergic dysfunction from the early stage of PD.
Ebina et al. 2024 [110]	Identify the clinical characteristics of patients with PD with dual SD compared with those with single SD.	Sympathetic denervation in major salivary glands and cardiac MIBG uptakes were significantly reduced in PD. Patients in the dual SD group were older and had more severe hyposmia and autonomic dysfunction, except for motor features.	Patients with PD with dual-sympathetic denervation have more severe non-motor features than other patients. Autonomic dysfunction might progress independently from dopaminergic degeneration.
Rasmussen et al. 2024 [111]	Assess the agreement between clinical cardiovascular adrenergic function and adrenergic innervation in T2D.	T2D patients had significantly lower early and delayed H/M ratios and lower WR. Lower total recovery and shorter pressure recovery time responses from the Valsalva maneuver were significantly correlated to lower H/M early and lower WR for Total Recovery.	There was impairment of sympathetic innervation in T2D patients based on parameters derived from MIBG cardiac scintigraphy (low early H/M, delayed H/M, and WR).
Sung et al. 2024 [112]	Compare the diagnostic performances of 18 F-FDOPA PET/CT and I-123 MIBG SPECT/CT for pheochromocytoma and paraganglioma (PPGL).	18 F-FDOPA PET/CT exhibited noninferior sensitivity (95.7%) compared with I-123 MIBG SPECT/CT (91.3%). 18 F-FDOPA PET/CT demonstrated significantly higher sensitivity compared with 123 I-MIBG SPECT/CT (86.2% vs. 65.5%).	18 F-FDOPA PET/CT demonstrated noninferior sensitivity and comparable specificity to I-123 MIBG SPECT/CT in diagnosing PPGL. 18 F-FDOPA PET/CT outperformed I-123 MIBG SPECT/CT in the assessment of PPGL recurrence and metastasis.
Ishibashi et al. 2023 [113]	I-123 MIBG for evaluation of the severity and prognosis of heart failure	Predischarge and 6-month post-discharge survival showed significant differences in groups	Prognostic model using I-123 MIBG scintigraphy was useful in predicting mortality risk in patients with heart failure.
Totsune et al. 2023 [114]	Identify subtypes of PD using nuclear imaging biomarkers targeting the cardiac sympathetic nervous and nigro-striatal systems	Three clusters were identified and showed distinct characteristics in onset ages and dopamine-replacement therapy and deep brain stimulation requirements.	Nuclear imaging biomarker-based classification can be used to identify clinically and pathologically relevant PD subtypes with distinct disease trajectories.
Conte et al. 2023 [115]	Evaluate sex differences of heart failures with prediction of cardiac arrhythmic events (AE).	No significant variables for prediction of AE were found in females. Early SS, late SS, ejection fraction, and late heart-to-mediastinum ratio were statistically significant predictors of AE in males.	I-123 MIBG represents a more effective tool for the prediction of AE in male patients than in women.
Zhou et al. 2023 [116]	Compare the diagnostic ability of minimal residual disease detection and I-123 MIBG SPECT/CT.	The diagnostic accuracy of planar and tomographic imaging was 3.5% and 95.8%, respectively.	I-123 MIBG SPECT/CT improves the diagnostic efficiency of neuroblastoma.
Ebina et al. 2023 [117]	Analyze I-123 MIBG uptake in parotid and submandibular glands in Parkinson’s disease (PD).	MIBG uptake ratio in the parotid glands/mediastinum (P/M) and submandibular glands/mediastinum (S/M) were significantly reduced in PD patients. Sensitivity and specificity were 54.8% and 59.1% for delayed phase P/M ratio; sensitivity and specificity were 59.5% and 61.0% for delayed phase S/M ratio, respectively.	MIBG uptake in parotid and submandibular glands was reduced PD. Sympathetic denervation in the major salivary glands and myocardium might progress independently.
Chun et al. 2023 [118]	Compared the diagnostic performances; investigated the optimal imaging protocol of I-123 MIBG cardiac scintigraphy in patients suspected of Parkinson’s disease.	Sensitivity, specificity, accuracy, PPV, and NPV of heart-to-mediastinum ratio and washout rate were maximized at 4 h PPVs persistently >92.7% at earlier time points and shorter time intervals.	4 h-delayed imaging is recommended for the best diagnostic performances in I-123 MIBG cardiac scintigraphy. Diagnostic performances to differentiate PD, PDD, and DLB from non-Parkinson’s diseases were suboptimal, but it can be used as an auxiliary measure.
Fedorova et al. 2023 [119]	Investigated thyroid I-123 MIBG uptake in patients with PD and DM	Reduced I-123 MIBG uptake was only observed in the PD group.	Both DM and PD patients showed decreased I-123 MIBG uptake in thyroid gland with the largest reduction seen in DM patients. This finding suggests that thyroid I-123 MIBG uptake is not a good marker to differentiate PD from diabetic patients.
Okuda et al. 2023 [120]	Developed a method of standardizing the heart-to-mediastinal ratio in I-123 MIBG images.	Conversion coefficients were superior when estimated by machine learning compared with classical multiple linear regression model. The experimental, MC-simulated, and ML-estimated conversion coefficients agreed, being, 0.54, 0.55, and 0.55 for low; 0.74, 0.70, and 0.72 for low–middle; and 0.88, 0.88, and 0.88 for medium energy collimators.	Machine-learning model estimated conversion coefficients without the need for phantom experiments.
Yasumoto et al. 2023 [121]	Evaluate myocardial SUV and assess its accuracy.	RCs and % CV of maximum SUVmax, and average SUVmaxs were 36.5% and 4.99%; 3.6% and 4.84%, respectively. RC and % CV of H/M ratio were 15.0% and 1.50%.	Myocardial SUV can provide quantitative values slightly closer to theoretical values than the H/M ratios.
Nakajo et al. 2023 [122]	Examine the usefulness of SUVmax of myocardial I-123 MIBG to characterize myocardial function.	LV diastolic dysfunction was inversely related to myocardial I-123 MIBG uptake.	Myocardial I-123 MIBG SUVmax may be useful for characterizing cardiac function in patients with pheochromocytoma.
Oh et al. 2023 [123]	Determine time to resolution of I-123 MIBG avidity after RT for neuroblastoma and local failure rate.	Median time to MIBG resolution after RT was 78 days.	Primary lesions without residual disease had excellent local control.
Gargiulo et al. 2023 [64]	Summarize applications of I-123 MIBG imaging in cardiac sympathetic neuronal function.	HF is frequently associated with comorbidities such as diabetes, obesity, and kidney disease that may affect cardiac adrenergic innervation.	Cardiac imaging with I-123 MIBG can be a useful tool to reduce morbidity and prevent adverse events in heart failure patients.
Mishkina et al. 2023 [124]	Assess significance of scintigraphic evaluation of cardiac sympathetic innervation and contractility to predict response to cardiac resynchronization therapy (CRT).	One-year follow-up post-CRT, I-123 MIBG uptake ratio was an independent predictor of CRT response in non-ischemic HF patients (OR 1.469; 95% CI 1.076–2.007, *p* = 0.003)	CRT response can be predicted by cardiac I-123 MIBG scintigraphy, specifically by heart-to-mediastinum ratio in non-ischemic HF and by mechanical dyssynchrony index HBW in ischemic HF.
Verschure et al. 2022 [68]	The role of I-123 MIBG imaging in chronic heart failure	Linear relationship between I-123 MIBG-derived parameters and overall prognosis.	Cardiac I-123 MIBG scintigraphy is a feasible technique to evaluate the global and regional cardiac sympathetic innervation.
Yadgarov et al. 2022 [125]	Evaluate the prognostic significance of parameters derived from pretherapeutic I-123 MIBG SPECT/CT in high-risk patients with neuroblastoma (NB).	Multivariable analysis identified high asphericity (≥65%) and metabolic tumor volume (≥50 mL) as the only factors associated with worse event-free survival.	Imaging parameters related to tumor metabolic activity, tumor asphericity, and MTV, provided prognostic value for event-free survival in high-risk NB patients.
Roberts et al. 2022 [65]	Compare early and delayed heart-to-mediastinum ratios (HMR) and make recommendations for clinical practice.	Histograms of early and delayed HMR showed two groups for delayed imaging. Accuracy results were higher for delayed imaging than early imaging (73 vs. 77%), sensitivity 63 vs. 65%, and specificity 82 vs. 88%.	A delayed image could be acquired only if the early result is borderline. This removes the need for delayed imaging in about 70% of patients and reduces the time patients have to wait.
Clement et al. 2022 [126]	Evaluate the prevalence of thyroid dysfunction in survivors of a neuroblastic tumor who received diagnostic I-123 MIBG only.	After a median follow-up of 6.6 years, thyroid function was normal in 95.8% of survivors. In 29.2% of the patients and 11.1% of images, I-123 uptake was visible in the thyroid.	Randomized controlled trials are required to investigate whether administration of I-123 MIBG without thyroid protection is harmful to the thyroid gland.
Lucas et al. 2022 [127]	Identify predictors of metastatic site failure (MSF) at new and/or original sites in high-risk neuroblastoma patients.	Multivariate CPHr identified inability to undergo transplant and/or maintenance chemotherapy and presence of lung metastases at diagnosis as predictors of new MSF. The new MSF-free survival rate at 3 years was 25% and 87% in patients with and without high-risk factors.	Persistence of MIBG avidity following induction chemotherapy and transplant at metastatic site increased the hazard for MSF.
Delgado-Silva et al. 2022 [128]	Assess cardiac sympathetic activity and investigate the role of myocardial I-123 MIBG scintigraphy for cardiovascular risk stratification of patients with resistant hypertension treated with renal denervation.	Early heart–mediastinum ratio (HMR) was significantly lower at baseline in responder group. Both the late HMR and the washout rate were identical, and no significant correlation between response to renal denervation or any MIBG imaging index was found.	Renal denervation effectively lowered blood pressure in the majority of patients, but I-123 MIBG was not useful for predicting the response.
Matsubara et al. 2022 [129]	Validate the diagnostic accuracy of I-123 MIBG myocardial scintigraphy for Lewy body diseases (LBD) against autopsy, the gold standard.	H/M ratio had a sensitivity and specificity of 70.0% and 96.2%. Delayed H/M ratio had a sensitivity and specificity of 80.0% and 92.3%. The washout rate had a sensitivity and specificity of 80.0% and 84.6%. The proportion of residual tyrosine hydroxylase–immunoreactive cardiac sympathetic fibers strongly correlated with the amount of cardiac I-123 MIBG uptake.	I-123 MIBG myocardial scintigraphy could differentiate LBDs from similar diseases. Abnormal I-123 MIBG myocardial scintigraphy findings strongly support the presence of LBD and cardiac sympathetic denervation.
Adaniya et al. 2022 [130]	Assess the association between a decrease in lung MIBG uptake with antidepressant intake and the myocardial MIBG uptake	All patients with decreased lung uptake were treated with antidepressants.	Antidepressants probably blocked MIBG uptake in lungs, and a remarkable decrease in lung uptake can be a signal to check a patient’s medication status.
Tamaki et al. 2022 [131]	Validate the risk model to predict post-discharge clinical outcomes in patients with acute decompensated heart failure (ADHF)	There was a significant difference in incidence of cardiac death among groups. The 2-year cardiac mortality risk model had a higher C-statistic for the prediction of cardiac mortality.	2-year MIBG-based cardiac mortality risk model is useful for predicting post-discharge clinical outcomes in patients with ADHF.
Park et al. 2022 [132]	Investigate the relationships of plasma α-synuclein levels with cardiac MIBG and striatal DAT uptake.	The plasma α-synuclein level correlated with early and delayed MIBG heart-to-mediastinum ratios, and its correlation with delayed H/M ratio remained significant after adjustment with age, disease duration, motor severity, and striatal DAT uptake.	Plasma α-synuclein levels more readily reflect the peripheral deposition of Lewy bodies than their central deposition.
Seo et al. 2022 [133]	Investigated the prognostic significance of residual MIBG-positive disease at each treatment phase.	Residual MIBG-positive disease at postinduction and post-tandem HDCT/auto-SCT evaluation was highly correlated with the risk of progression.	Residual MIBG-positive disease during treatment predicted unfavorable outcomes in high-risk neuroblastoma, even under tandem HDCT/auto-SCT.
Nitta et al. 2022 [134]	Investigate factors influencing CSN activity of patients with severe aortic stenosis	H/M ratio was 3.1 ± 0.5, delayed H/M ratio was 2.8 ± 0.6, and WR was 35% ± 13%.	Coronary artery disease is an independent predictor of delayed H/M ratio, and aortic valve area is an independent predictor of WR in severe AS.
Vancraeynest et al. 2022 [135]	Assess concordance of I-123 MIBG and microscopic BM examination for detecting BM involvement at diagnosis and before autologous stem cell collection (ASCC).	Concordance rates for I-123 MIBG versus microscopic BM examination were acceptable (77.1% and 85.3%). The concordance rate for biopsy versus aspirate at diagnosis was 80.6%.	I-123 MIBG scintigraphy and a microscopic examination of BM should be used as complementary tools in the evaluation of BM involvement.
Seo et al. 2022 [136]	Elucidate the prognostic value of cardiac sympathetic nerve dysfunction using I-123MIBG SPECT imaging in heart failure with preserved left ventricular ejection fraction.	Total defect score (TDS) was significantly associated with cardiac events after multivariate Cox adjustment. Patients with high TDS levels had a significantly greater risk of cardiac events.	Cardiac I-123 MIBG SPECT imaging provided useful prognostic information in nonischemic ADHF patients with HFpEF.
Kadoya et al. 2022 [137]	Examine whether TAVR-dependent early improvements in sympathetic nervous function are transitory or sustainable.	Multivariate analysis revealed mPG as an independent predictor of mid-term improvement in the late H/M. Patients with a high baseline mPG (≥58 mmHg) exhibited a significantly greater increase in the late H/M.	Cardiac sympathetic nervous function demonstrated sustained improvement from within 2 weeks after transcatheter aortic valve replacement (TAVR) until 6 to 12 months later.
Kitamura et al. 2022 [138]	Introduce a new semi-quantitative analysis using I-123 MIBG SPECT/CT, tumor-to-liver count ratio, and tumor-to-muscle count ratio.	The AUC for T/Mmax was significantly higher than those of the planar score and SPECT score by ROC analysis.	Semi-quantitative value of I-123 MIBG SPECT/CT is more useful than conventional visual evaluation for differentiating pheochromocytomas from cortical adenomas.
Kobayashi et al. 2021 [139]	Estimate gastrointestinal tract absorption of cationic anticancer drugs and medicines via whole-body imaging following oral I-123 MIBG administration.	Biodistribution analyses and SPECT imaging studies showed significantly lower accumulation of I-123 MIBG in blood, heart, liver, and bladder in DSS-induced experimental colitis mice and mice with cimetidine loading, whereas significantly higher stomach and kidney accumulations were observed after I-123 MIBG injection.	Imaging after oral administration can be used to estimate gastrointestinal absorption in small intestine via OCTN and/or OCT by measuring radioactivity in the heart, liver, and bladder.
Kumakura et al. 2021 [140]	Optimized indices that capture norepinephrine kinetics, tested their diagnostic performance, and determined I-123 MIBG performance.	MIBG loss was highly discriminative, particularly for patients with low myocardial I-123 MIBG trapping, and the new indices outperformed existing ones.	I-123 MIBG turnover can be quantified in 30 min using a three-parameter model based on I-123 MIBG time–activity curves.
Lange et al. 2021 [141]	Study left ventricular cardiac sympathetic activity in patients treated with pulmonary vein isolation (PVI) and antiarrhythmic drugs.	PVI with point-by-point radiofrequency ablation led to a significantly (*p* < 0.05) higher visual sympathetic innervation defect score when comparing pre- and post-PVI. Differences in cardiac sympathetic innervation remodeling following PVI suggest an important role of cardiac autonomous nervous system in the maintenance of sinus rhythm following PVI.	PVI results in novel defects of cardiac sympathetic innervation.
